# The Role Of Parafacial Neurons In The Control Of Breathing During Exercise

**DOI:** 10.1038/s41598-017-17412-z

**Published:** 2018-01-10

**Authors:** Alla Korsak, Shahriar Sheikhbahaei, Asif Machhada, Alexander V. Gourine, Robert T. R Huckstepp

**Affiliations:** 1 Centre for Cardiovascular and Metabolic Neuroscience, Neuroscience, Physiology and Pharmacology, University College London, London, WC1E 6BT United Kingdom; 2 School of Life Sciences, University of Warwick, Coventry, CV4 7AL United Kingdom

## Abstract

Neuronal cell groups residing within the retrotrapezoid nucleus (RTN) and C1 area of the rostral ventrolateral medulla oblongata contribute to the maintenance of resting respiratory activity and arterial blood pressure, and play an important role in the development of cardiorespiratory responses to metabolic challenges (such as hypercapnia and hypoxia). In rats, acute silencing of neurons within the parafacial region which includes the RTN and the rostral aspect of the C1 circuit (pF_RTN/C1_), transduced to express HM_4_D (G_i_-coupled) receptors, was found to dramatically reduce exercise capacity (by 60%), determined by an intensity controlled treadmill running test. In a model of simulated exercise (electrical stimulation of the sciatic or femoral nerve in urethane anaesthetised spontaneously breathing rats) silencing of the pF_RTN/C1_ neurons had no effect on cardiovascular changes, but significantly reduced the respiratory response during steady state exercise. These results identify a neuronal cell group in the lower brainstem which is critically important for the development of the respiratory response to exercise and, determines exercise capacity.

## Introduction

**Figure 1:**
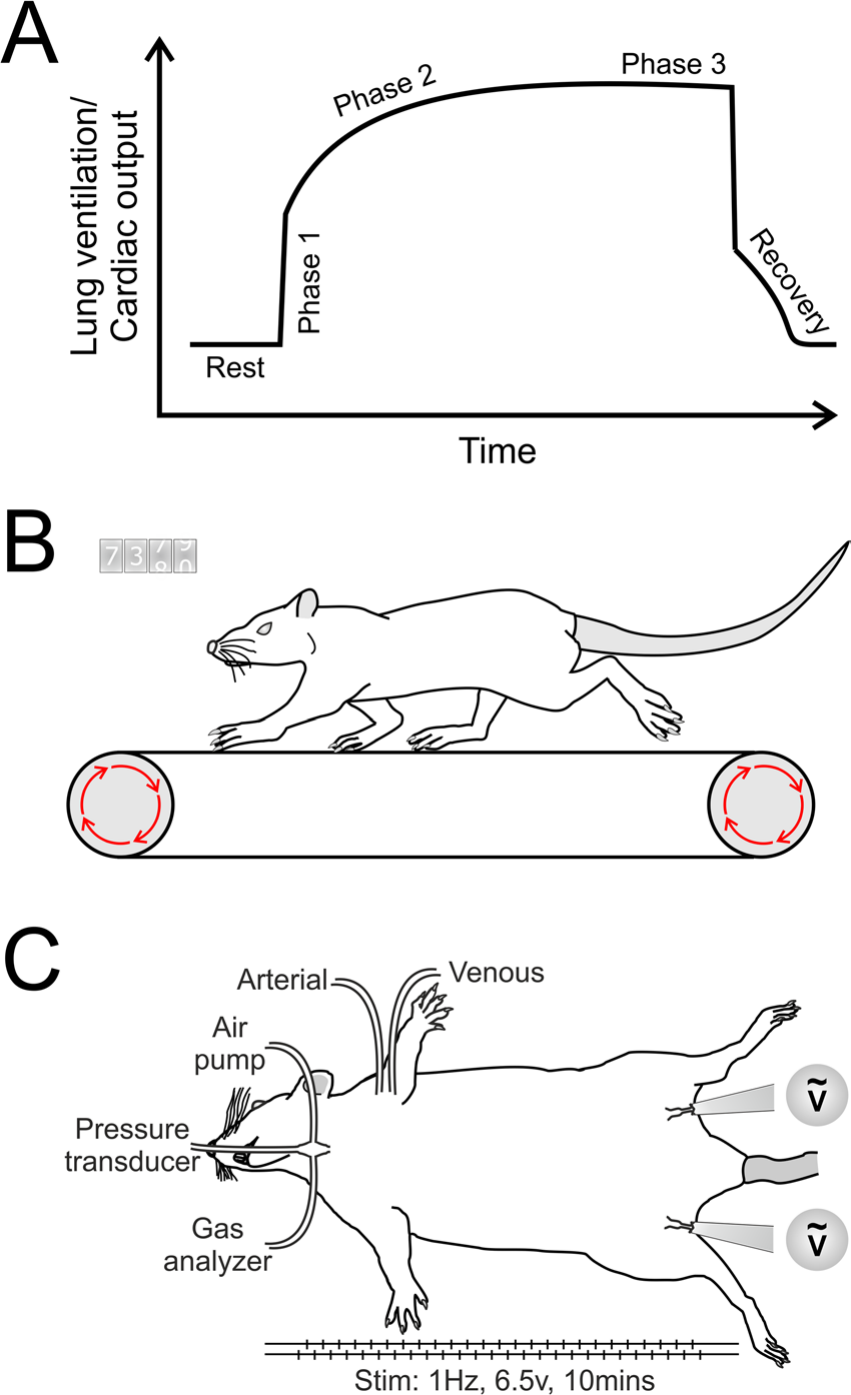
Phases of exercise and general experimental design. (**A**) Schematic drawing illustrating 3 phases of the cardiovascular and respiratory responses to exercise. (**B**) Treadmill running paradigm to determine exercise capacity in rats. (**C**) Experimental paradigm of simulated exercise involving electrical stimulation of femoral or sciatic nerves in urethane-anaesthetised rats to study exercise-induced cardiovascular and respiratory responses.

Cardiovascular and respiratory activities rapidly adapt to ever-changing behavioural and environmental conditions to ensure an appropriate supply of metabolic substrates to, and removal of metabolic waste products from, all tissues of the body. To match oxygen and glucose delivery with increased metabolic demands during exercise, ventilation and cardiac output are rapidly adjusted by several physiological feedforward and feedback mechanisms. In response to exercise, cardiorespiratory changes develop in 3 distinct phases (Fig. 1A). Phase 1: an initial rapid increase in cardiorespiratory output that precedes increases in oxygen demand; Phase 2: a continuing gradual increase in ventilation and cardiac output; Phase 3: a plateau phase of elevated cardiorespiratory output, as steady state level of exercise is reached^1–3^. The rapid increase in cardiorespiratory output (Phase 1) occurs too soon after the onset of exercise to involve metabolic feedback, and can even occur prior to exercise^4^. During Phase 1, descending cortical feed-forward and/or muscle reflex mechanisms increase ventilation and cardiac output in anticipation of, and during the initial phases of, exercise^5–9^. Feedback signals from muscle stretch/metabolic receptors and possibly central and peripheral chemoreceptors are responsible for the gradual increases in (Phase 2), and maintenance of (Phase 3), enhanced cardiorespiratory activity during continued exercise^10–15^.

In the parafacial region of the rostral ventrolateral medulla oblongata a distinct group of neurons which reside within the retrotrapezoid nucleus (RTN) control breathing by integrating chemosensory information received from other CNS structures^16–19^, local astrocytes^20–22^, and the peripheral chemoreceptors^16,23,24^. RTN neurons may themselves be sensitive to changes in local parenchymal PCO_2_/pH^25^ and were proposed to play an important role in the mechanisms of central respiratory CO_2_ chemosensitivity^26,27^. There is also evidence that the RTN neurons are critically important for the recruitment of active expiratory activity^28–30^, and that these neurons are activated during exercise^31^.

Another notable brainstem neuronal population, which resides in close proximity to the RTN, is the C1 catecholaminergic cell group which contributes to the generation of central sympathetic drive and is critically important for cardiovascular control in conditions of increased metabolic demand^27,32^.

We hypothesised that these two important neuronal populations orchestrate the cardiovascular (C1 neurons) and respiratory (RTN neurons) responses to exercise and, by doing so, determine exercise capacity. To test this hypothesis, we used adeno-associated viral (AAV) vectors to transduce the parafacial brainstem region which includes the RTN and the rostral aspect of the C1 circuit (pF_RTN/C1_) to express HM_4_D (G_i_-DREADD) receptor (HM_4_DR) allowing rapid, acute and reversible silencing of targeted neuronal populations in experimental rats. We determined the effect of inhibiting this region on exercise capacity (Fig. 1B), and cardiovascular and respiratory responses to simulated exercise (electrical sciatic or femoral nerve stimulation in urethane anaesthetised spontaneously breathing rats; Fig. 1C).

## Results

### Targeting pF_RTN/C1_ neurons to express HM_4_DR

**Figure 2:**
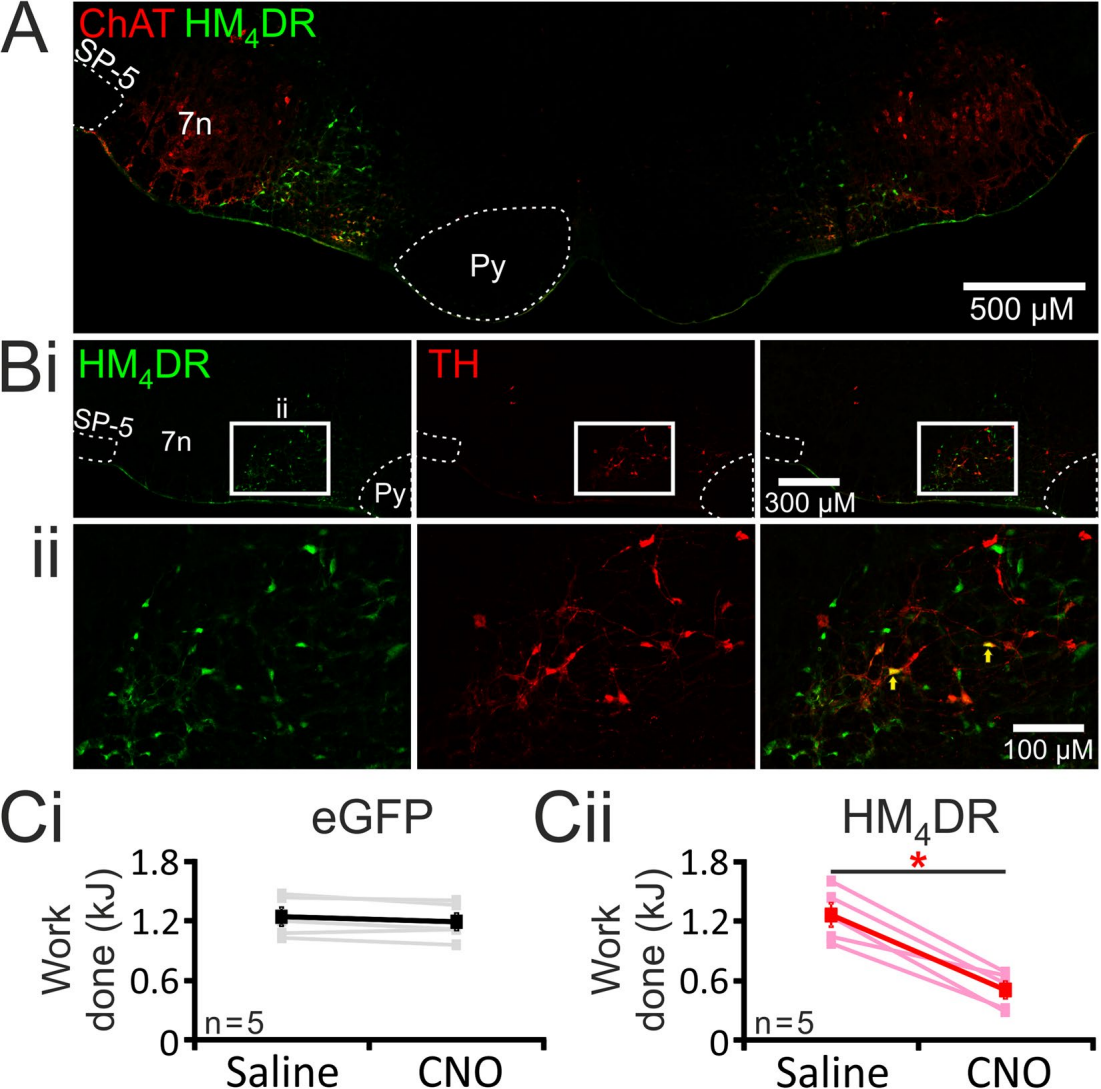
Reduced exercise capacity in conditions of pF_RTN/C1_ inhibition in rats. (**A**, **B**) Histological analysis of virally-induced HM_4_DR expression in the parafacial region of the rostral ventrolateral medulla oblongata. (**A**) Neurons transduced to express HM_4_DR (identified by mCitrine immunoreactivity, green) are concentrated medial and ventral to the facial nucleus, identified by ChAT immunostaining (red). (**B**) Neurons transduced to express HM_4_DR/ mCitrine (green), are interspearsed with catecholaminergic C1 neurons (identified by TH immunoreactivity, red). A proportion of C1 neurons were found to be transduced (yellow: arrows). Anatomical landmarks (dashed white lines) are shown to aid illustration of the injection sites, 7n – facial nucleus, Py – pyramidal tract, SP-5 – spinal trigeminal tract. (**Ci**) Exercise capacity is unaffected by CNO (2 mg·kg^−1^) in rats transduced to express eGFP by the pF_RTN/C1_ neurons; (**Cii**) Exercise capacity is markedly reduced by CNO (2 mg·kg^−1^) in rats transduced to express HM_4_DR by the pF_RTN/C1_ neurons.

As expected, the morphology of cells expressing the transgenes indicated that only neurons were transduced, however not all neurons within the injection site were transfected; for example neurons of the facial nucleus were unaffected. Based on the locations of the injection sites and immunostaining, all neurons expressing the transgenes were found to lay within the RTN and C1 areas of the rostral ventrolateral medulla oblongata. RTN neurons are located medial and ventral to the facial nucleus (identified using ChAT immunostaining); C1 neurons (identified using TH immunostaining) partially overlap with, and extends caudally from, the RTN (Fig. 2).

#### Treadmill exercise experiments

In representative sections of five rat brainstems targeted to express HM_4_D receptor 124 ± 9 neurons per side were found to express mCitrine and, therefore, the HM_4_D receptor. No mCitrine-positive neurons were found to express TH indicating that in this group of animals no C1 neurons were transduced to express the HM_4_DR. In three brains, a small number of ChAT-positive cells were found to express the HM_4_DR (4 ± 2 neurons per side). These neurons have previously been shown to modulate non-chemosensory information^33^. The majority of transduced HM_4_DR-expressing neurons (119 ± 8 per side), were considered to be parafacial RTN neurons based on their anatomical location in relation to the facial nucleus^28,34^. No labelling of neurons in regions of the medulla oblongata other than within the parafacial region was found.

#### Simulated exercise experiments

In nine rat brainstems targeted to express HM_4_D receptor, 255 ± 41 neurons per side were found to express mCitrine and, therefore, the HM_4_D receptor. In five brainstems, 55 ± 14 C1 neurons (expressing TH) were identified per side. A significant number of these neurons were transduced to express the HM_4_DR (identified by expression of mCitrine; 22 ± 5 neurons per side; n = 5). Thus, 44 ± 6% of identified C1 neurons within the injection site were found to be transduced. The remaining majority of HM_4_DR-expressing neurons (that did not express TH) were considered to be RTN neurons based on their anatomical location in relation to the facial nucleus^28,34^. In the brainstems of three rats a small number (25 ± 9 neurons per side) of non-chemosensory ChAT positive cells^33^ were found to be transduced. The cardiorespiratory responses to simulated exercise in these three rats did not differ from the larger cohort. No labelling of neurons in regions of the medulla oblongata other than within the parafacial region was found.

### Silencing of the pF_RTN/C1_ neurons impairs exercise capacity

Exercise capacity of rats transduced to express HM_4_DR (n = 5) or eGFP (n = 5) in the pF_RTN/C1_ neurons was determined using an exercise model on a rodent treadmill. After the initial training period, following injections of saline, rats expressing HM_4_DR and eGFP achieved similar baseline exercise capacities (p = 0.9; Fig. 2C). Exercise capacity in control animals expressing eGFP was unaffected by administration of CNO (control: 1.2 ± 0.2 vs CNO:1.2 ± 0.2 kJ: p = 0.1; Fig. 2Ci). Inhibition of the pF_RTN/C1_ neurons expressing HM_4_DR following CNO administration dramatically reduced exercise capacity (by ~60%; from 1.3 ± 0.1 to 0.5 ± 0.1 kJ; p < 0.002; Fig. 2Cii). These data suggested that the activity of pF_RTN/C1_ neurons determines the ability to exercise.

### Silencing of the pF_RTN/C1_ neurons reduces the respiratory response to simulated exercise

In a rat model of simulated exercise (electrical stimulation of the sciatic or femoral nerve in urethane-anaesthetised spontaneously breathing rats), we next determined whether impaired exercise capacity in conditions of pF_RTN/C1_ silencing is due to a reduction in the respiratory and/or cardiovascular responses.

**Figure 3:**
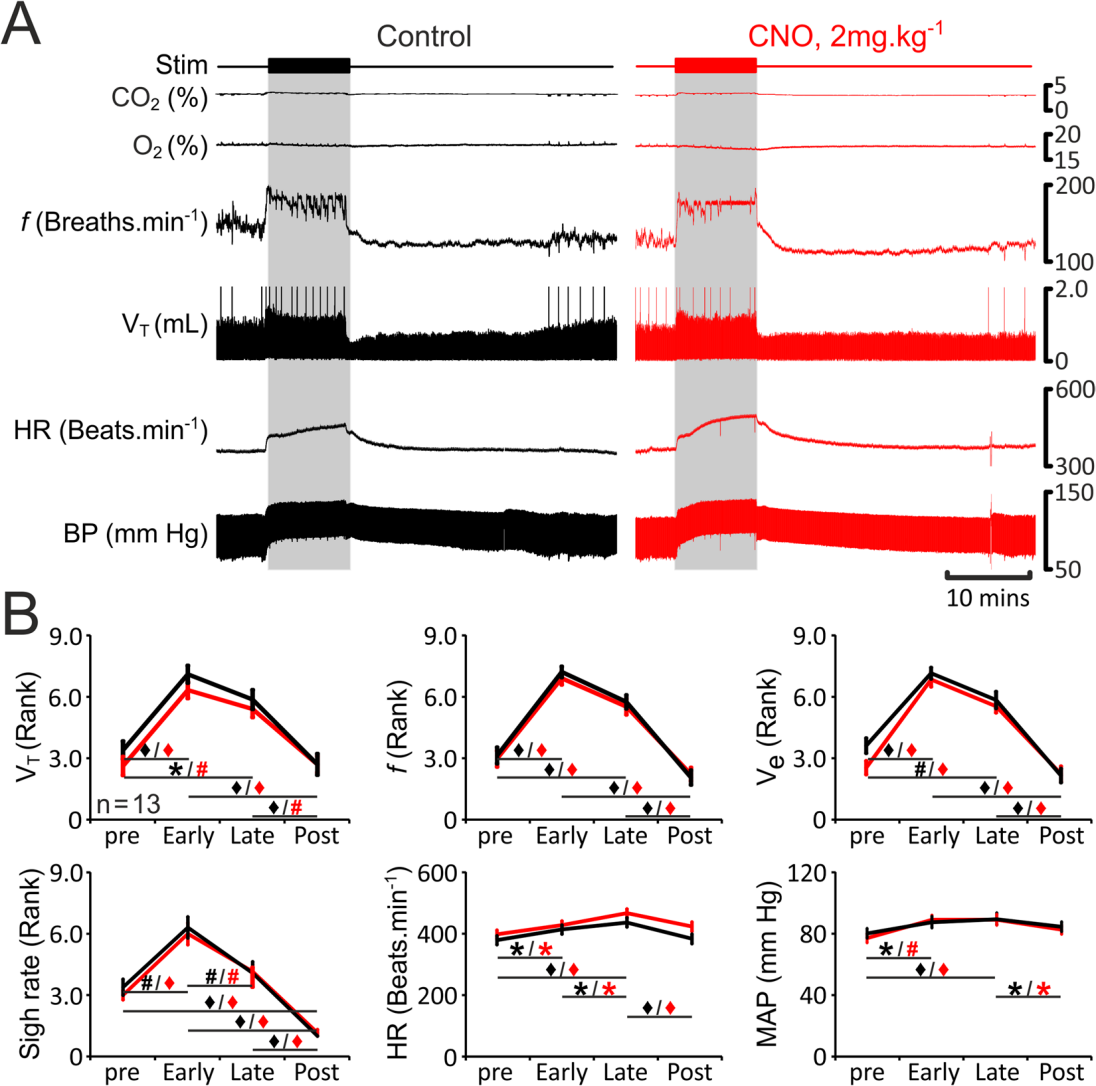
Cardiorespiratory responses to exercise in anaesthetized rats transduced to express eGFP by the pF_RTN/C1_ neurons are not affected by CNO. (**A**) Raw data illustrating changes in end-tidal CO_2_, end-tidal O_2_, respiratory frequency (*f*), tidal volume (V_T_), heart rate (HR) and the arterial blood pressure (BP) induced by simulated exercise (sciatic nerve stimulation: 6.5 v, 100 ms, 1 Hz, 10 mins) before (control, black) and after administration of CNO (2 mg·kg^−1^; red) in a rat transduced to express eGFP by the pF_RTN/C1_ neurons. Sigh signals are truncated so that tidal volume changes could be seen more clearly. (**B**) Summary data (mean ± SEM) illustrating changes in V_T_, f, V_E_, sigh rate, HR and mean arterial blood pressure (MAP) during simulated exercise before (black) and after (red) CNO administration in rats transduced to express eGFP by the pF_RTN/C1_ neurons. Note there are no markers above the group data as no significant differences between the conditions, i.e., control vs CNO, were found. Markers below the group data indicate significant differences within conditions, i.e., baseline levels before the stimulation (pre), during the initial response to stimulation (Early), during the continued response to stimulation (Late), and after the response (Post): Black markers depict significant differences within the control condition and red markers indicate the significant differences in conditions of systemic CNO treatment, black horizontal lines show which comparisons are being made. ^*^p < 0.05, ^#^p < 0.01, ^♦^p < 0.001.

**Figure 4:**
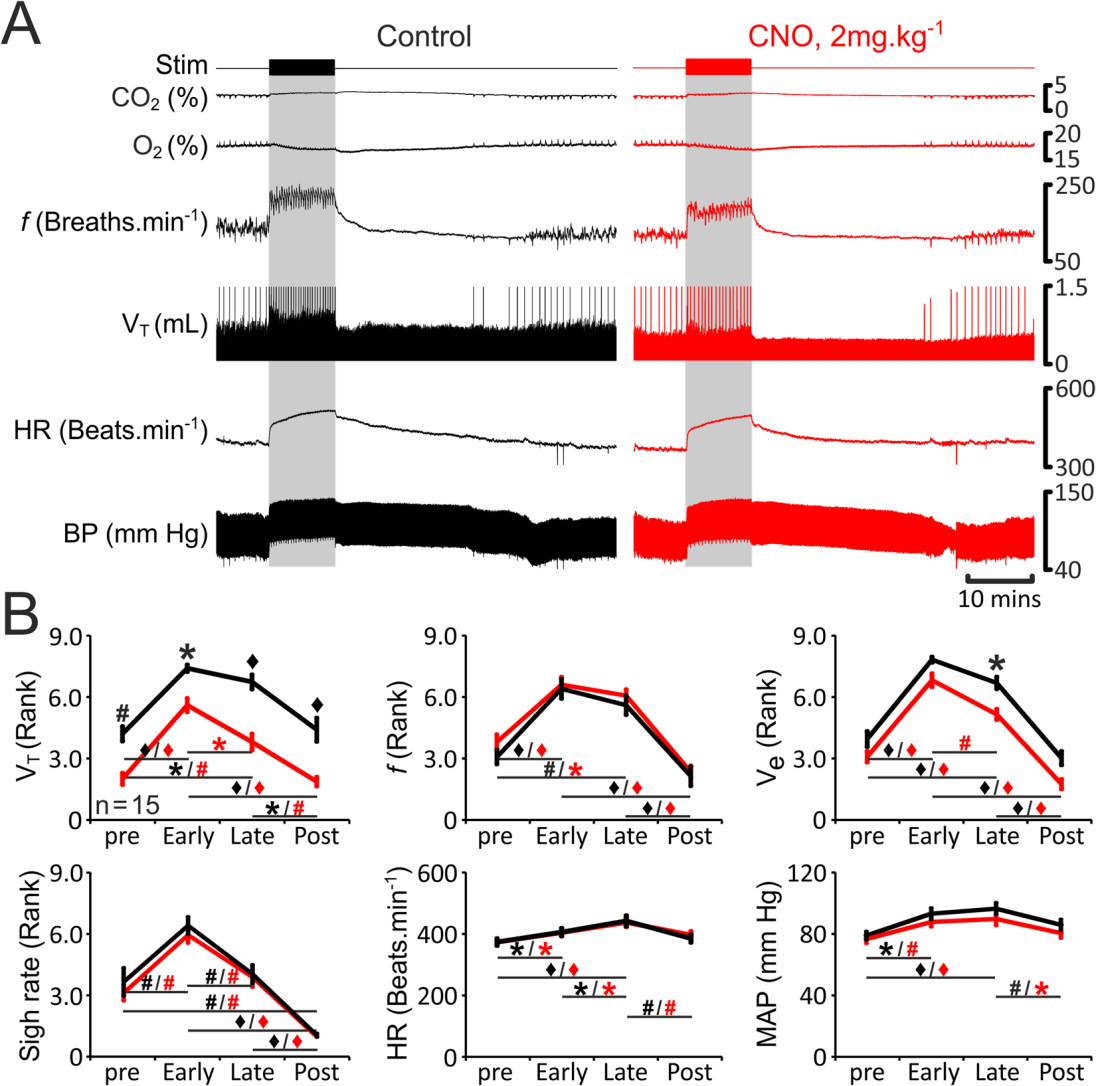
Reduced respiratory response to simulated exercise in conditions of pF_RTN/C1_ inhibition in anaesthetized rats. (**A**) Raw data illustrating changes in end-tidal CO_2_, end-tidal O_2_, *f*, V_T_, HR and BP induced by simulated exercise (sciatic nerve stimulation: 6.5 v, 100 ms, 1 Hz, 10 mins) in a rat transduced to express HM_4_DR by the pF_RTN/C1_ neurons before (control, black) and after administration of CNO (2 mg·kg^−1^; red). Sigh signals are truncated so that tidal volume changes could be seen more clearly. (**B**) Summary data (mean ± SEM) illustrating changes in V_T_, f, V_E_, sigh rate, HR and MAP during simulated exercise before (black) and after (red) CNO administration in rats transduced to express HM_4_DR by the pF_RTN/C1_ neurons. Markers above the group data depict significant differences between the conditions, i.e., control vs CNO. Markers below the group data indicate significant differences within conditions, i.e., baseline levels before the stimulation (pre), during the initial response to stimulation (Early), during the continued response to stimulation (Late), and after the response (Post): Black markers depict significant differences within the control condition and red markers indicate the significant differences in conditions of systemic CNO treatment, black horizontal lines show which comparisons are being made. *p < 0.05, ^#^p < 0.01, ^♦^p < 0.001.

In rats transduced to express eGFP (n = 13; Fig. 3) or HM_4_DR (n = 15; Fig. 4) in pF_RTN/C1_ neurons, simulated exercise triggered rapid increases in tidal volume (V_T_: 1.1 IQR 0.4 to 1.5 IQR 0.7 mL), respiratory frequency (*f*: 140 IQR 24 to 169 IQR 31 breaths·min^−1^), and minute ventilation (V_e_: 145 IQR 82 to 279 IQR 131 mL·min^−1^) (Figs 3 and 4). V_T_, *f*, and V_e_ remained elevated during the whole period of stimulation (V_T_: 1.4 IQR 0.6 mL, *f*: 158 IQR 31 breaths·min^−1^, V_e_: 192 IQR 87 mL·min^−1^) and gradually decreased back to baseline levels after the stimulation (V_T_: 0.9 IQR 0.5 mL, *f*: 128 IQR 28 breaths·min^−1^, V_e_: 122 IQR 34 mL·min^−1^) (Figs 3 and 4). Sigh rate increased at the onset of exercise (1 IQR 1 to 3 IQR 1 sighs·min^−1^), but was not sustained at this level during the whole period of stimulation (2 IQR 1 sighs·min^−1^) (Figs 3 and 4). Generation of sighs was abolished after the termination of stimulation (0 IQR 0 sighs·min^−1^; Figs 3 and 4). Electrical stimulation of the sciatic or femoral nerve was also associated with significant increases in HR (baseline: 379 IQR 84, early response: 414 IQR 57, maintained response: 436 IQR 21 beats·min^−1^) and MAP (baseline: 80 IQR 3, early response: 88 IQR 3, maintained response: 89 IQR 3 mmHg) (Figs 3 and 4). HR and MAP decreased to baseline values after the end of stimulation (383 IQR 56 beats·min^−1^, and 85 IQR 3 mmHg, respectively) (Figs 3 and 4). Interestingly, *f*, HR and MAP took a similar time to recover after the termination of simulated exercise (24 ± 4 mins, 24 ± 4 mins, and 23 ± 3 mins, respectively; n = 15). However, V_T_ returned to basal levels earlier, and was accompanied by re-initiation of sighing (15 ± 3 mins and 16 ± 3 mins, respectively; n = 15). In general, cardiorespiratory response to simulated exercise followed a temporal profile similar to that usually observed during ‘natural’ exercise (Fig. 1A). Furthermore, during electrical stimulation of the sciatic or femoral nerve expired CO_2_ increased whilst expired O_2_ decreased (Figs 3A and 4A) as is commonly reported during exercise^35–37^. Thus, this model of simulated exercise appears to accurately replicate mild-moderate exercise in animals and humans.

Cardiorespiratory responses to simulated exercise were similar in rats transduced to express HM_4_DR or eGFP in the pF_RTN/C1_ neurons suggesting that HM_4_DR expression *per se* (i.e. in the absence of a ligand) has no effect on the activity of the targeted neurons. Administration of CNO had no effect on the respiratory and cardiovascular responses triggered by simulated exercise in rats expressing eGFP in the pF_RTN/C1_ area (n = 13; Fig. 3). Therefore, the response was found to be consistent over time and was unaffected by CNO.

Changes in sigh rate, HR and MAP recorded during simulated exercise were unaffected following inhibition of the pF_RTN/C1_ (Fig. 4). However, silencing of the transduced population of pF_RTN/C1_ neurons had a profound effect on changes in V_T_ induced by simulated exercise: V_T_ was reduced at baseline and during all phases of exercise (Fig. 4). Although pF_RTN/C1_ inhibition had no effect on changes in *f*, V_e_  was significantly reduced during the late phase of simulated exercise (Fig. 4). These data suggest that the inability to mount and maintain an appropriate tidal volume response to match ventilation with metabolic demand during the steady state phase of exercise is the likely cause of impaired exercise capacity in conditions of pF_RTN/C1_ silencing.

### Silencing of the pF_RTN/C1_ neurons reduces the respiratory and cardiovascular responses to CO_2_

RTN neurons are believed to mediate respiratory responses to hypercapnia^28,38–40^, whereas C1 neurons are not thought to respond to changes in *P*CO_2_/pH^27,41^. To functionally verify successful targeting of a significant proportion of pF_RTN/C1_ neurons, we next determined the effect of silencing of transfected neurons on CO_2_-induced respiratory and cardiovascular responses in urethane-anaesthetised spontaneously breathing rats.

**Figure 5:**
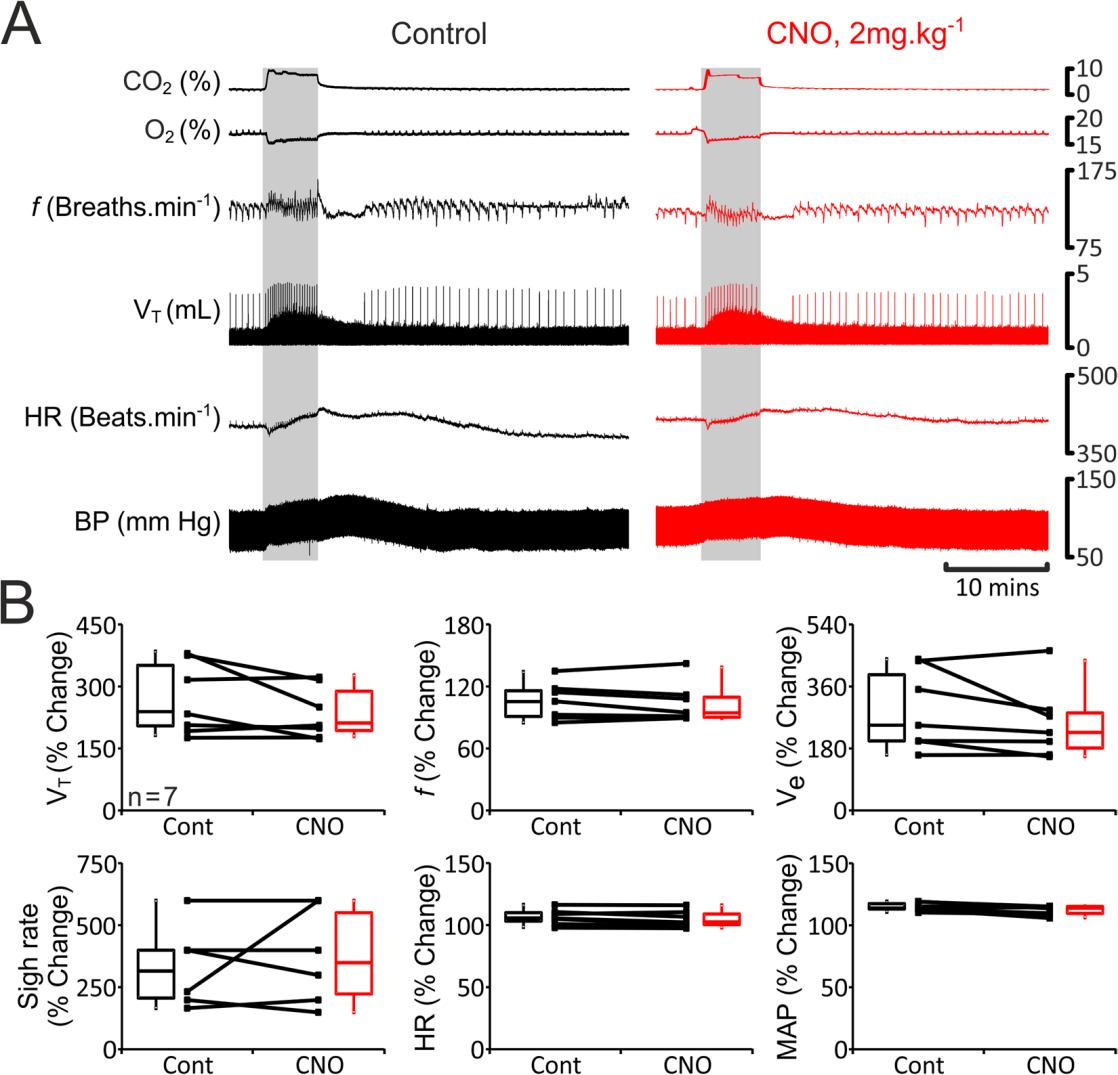
Cardiorespiratory responses to hypercapnia in anaesthetized rats transduced to express eGFP by the pF_RTN/C1_ neurons are not affected by CNO. (**A**) Raw data illustrating changes in end-tidal CO_2_, end-tidal O_2_, *f*, V_T_, HR and BP induced by systemic hypercapnia (gray boxes) in a rat transduced to express eGFP by the pF_RTN/C1_ neurons before (control, black) and after administration of CNO (2 mg·kg^−1^; red). (**B**) Summary data (mean ± SEM) illustrating magnitude of changes in V_T_, f, V_E_, sigh rate, HR and MAP induced by systemic hypercapnia in rats transduced to express eGFP by the pF_RTN/C1_ neurons before (black) and after (red) CNO administration.

**Figure 6:**
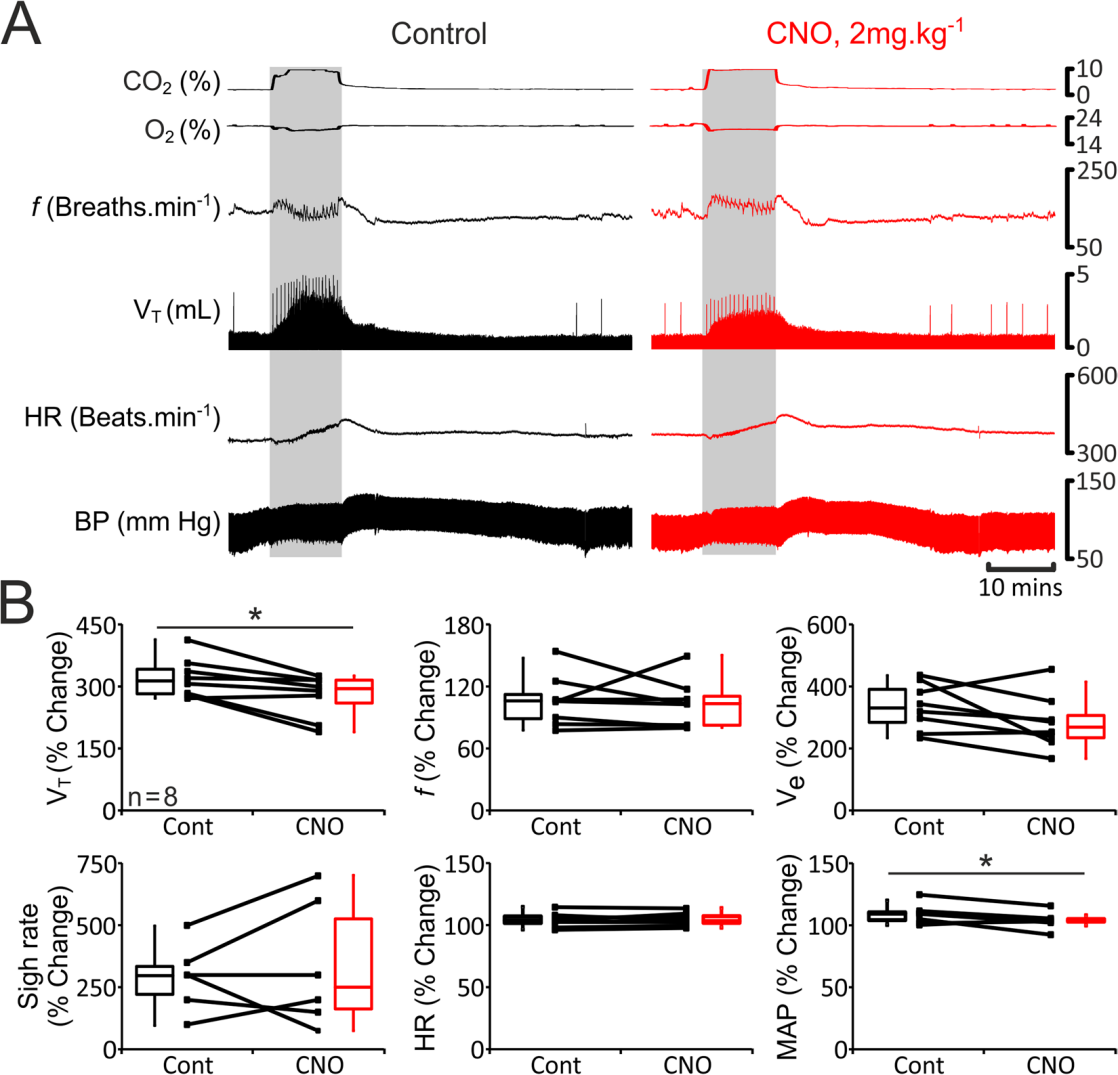
Reduced tidal volume and systemic arterial blood pressure responses to hypercapnia in conditions of pF_RTN/C1_ inhibition in anaesthetized rats. (**A**) Raw data illustrating changes in end-tidal CO_2_, end-tidal O_2_, *f*, V_T_, HR and BP induced by systemic hypercapnia (gray boxes) in a rat transduced to express HM_4_DR by the pF_RTN/C1_ neurons before (control, black) and after administration of CNO (2 mg.kg^−1^; red). (**B**) Summary data (mean ± SEM) illustrating magnitude of changes in V_T_, f, V_E_, sigh rate, HR and MAP induced by systemic hypercapnia in rats transduced to express HM_4_DR by the pF_RTN/C1_ neurons before (black) and after (red) administration of CNO. Sighing was absent in 2 rats at rest, thus n = 6 for this parameter. *p < 0.05.

In rats transduced to express eGFP in pF_RTN/C1_ neurons (n = 7; Fig. 5), hypercapnia increased V_T_ (1.3 IQR 0.2 to 3.0 IQR 2.5 mL), V_e_ (169 IQR 74 to 530 to 81 mL·min^−1^), sigh rate (0.6 IQR 1.2 to 3 IQR 1.2 sighs·min^−1^) and MAP (75 IQR 8 to 88 IQR 9 mmHg), but had no effect on *f* (131 IQR 24 to 138 IQR 34 breaths·min^−1^) and HR (402 IQR 70 to 425 IQR 47 beats·min^−1^; Figs 5 and 6). Cardiorespiratory responses to the increases in the level of inspired CO_2_ were found to be similar in rats transduced to express HM_4_DR (n = 8; Fig. 6) in pF_RTN/C1_ neurons. Administration of CNO had no effect on CO_2_-induced respiratory and cardiovascular responses in rats expressing eGFP (n = 7; Fig. 5). Thus, the response to systemic hypercapnia was consistent over the time of the experiment and was unaffected by CNO. In conditions of pF_RTN/C1_ inhibition, baseline V_T_ was reduced during the CO_2_ trials (p = 0.008), and CO_2_-induced increases in V_T_ and MAP were significantly smaller (n = 8; V_T_: by 19% from 313 ± 59 to 294 ± 55%, p = 0.03; and MAP: by 7%, from 110 ± 6 to 103 ± 3%, P = 0.04; Fig. 6). The magnitude of the observed effect of pF_RTN/C1_ inhibition on CO_2_-induced respiratory response was similar to that reported previously from the experiments when specific promoters were used to target RTN neurons^38^, indicating that the injections successfully transduced a significant proportion of RTN neurons involved in central respiratory CO_2_ chemosensitivity.

## Discussion

Physical exertion is essential for the survival of all animals, whether it be to avoid predators, escape natural disasters, or any other circumstances that require explosive power or endurance. Large increases in metabolic demand associated with enhanced muscular effort, require facilitated delivery of oxygen and metabolic substrates, and removal of waste products, e.g. CO_2_ and lactate. Mammals have developed intricate physiological feedforward and feedback mechanisms to ensure that metabolic demands of working muscles are met. Here, we tested the hypothesis that functional neuronal groups which reside in the parafacial region of the brainstem, including RTN and C1 cell populations, orchestrate cardiorespiratory responses during exercise.

C1 neurons provide an important source of central sympathetic drive and are critically important for cardiovascular control^27^. There is also evidence that C1 neurons may contribute to respiratory control^41,42^. In these experiments, reported changes in phrenic nerve activity consisted of 2 distinct components: an abrupt increase associated with augmented sympathetic activity and a lower amplitude sustained response^42^. Following lesioning of the C1 region the increased phrenic nerve activity associated with sympathetic nerve activity was abolished, whilst the lower amplitude response was preserved^42^. These data suggest that C1 neurons play only a minor role in the control of breathing (which appears to be secondary to their role in autonomic control). There is strong evidence that changes in blood pressure, are abolished by lesioning of the C1 region^42^, confirming that these neurons indeed play a critical role in cardiovascular control^27,43^.

In contrast, RTN neurons do not directly control systemic arterial blood pressure^44^, but provide a powerful drive to breathe at rest^28,40^, during systemic hypercapnia^22,25,38,39,45^, or when brain lactate concentration increases^46^. The RTN receives inputs from the peripheral respiratory chemoreceptors, other functional respiratory neuronal groups of the brainstem^47^, and, therefore, was proposed to function as an integrative centre of respiratory control^16–18^.

In this study, a sufficient number of parafacial neurons was transduced to result in a functionally significant reduction in output from both the RTN and C1 regions in anaesthetised rats (Figs 5 and 6). pF_RTN/C1_ inhibition had no effect on cardiovascular responses induced by simulated exercise (Figs 3 and 4), although a critical number of C1 neurons was affected to reduce the pressor response to CO_2_ (Figs 5 and 6). These data suggest that C1 neurons are not responsible for the development of cardiovascular response during exercise. Moreover, since dramatic (by 60%) reduction in exercise capacity following CNO administration was observed in conditions when parafacial neurons other than C1 cells were transduced to express HM_4_DR, we will now only discuss the observed effects as resulting from inhibition of the RTN neuronal population.

Experimental reduction or blockade of RTN neuronal activity (by lowering excitability, acute neuronal silencing, or lesioning of the region) decreases tidal volume or completely abolishes phrenic nerve activity at resting conditions^28,38,48,49^ and reduces the magnitude of the reflex increases in tidal volume induced by chemosensory challenges^28,38,40^. However, stimulation of the RTN neurons (optogenetic or pharmacological) increases the respiratory rate^34,44^. In this study, we found that in conditions of pF_RTN/C1_ silencing, the reduction of the respiratory response to CO_2_ was exclusively due to smaller increases in tidal volume, whilst increases in the respiratory rate were unaffected. These data are in agreement with the results of earlier studies suggesting that the RTN provides a powerful CO_2_-sensitive drive to breathe which primarily controls tidal volume^28,40^.

In conditions of pF_RTN/C1_ inhibition, tidal volume was reduced during all phases of simulated exercise. Inhibition of pF_RTN/C1_ neurons had variable effects on changes in the respiratory rate, such that minute ventilation during pF_RTN/C1_ inhibition was found to be significantly reduced only during the steady state phase of exercise (phase 3). Since ventilation during the initial phase of exercise (phase 1) was not affected, the RTN does not appear to receive/process feedforward inputs responsible for rapid increases in the respiratory activity at the onset of physical exertion. The importance of RTN only becomes apparent when the maintenance of enhanced respiratory effort is reliant on feedback mechanisms^6–9^. This explains why in conditions of pF_RTN/C1_ inhibition experimental animals were able to begin, but not to maintain, physical activity. Whilst a significant number of pF_RTN/C1_ neurons were transduced, the neurons involved in sigh generation^50^ either did not show tropism for the viral vector used or an insufficient number of these neurons were transduced to have an effect on sigh-generating circuits as a whole, as pF_RTN/C1_ inhibition had no effect on sigh rate either at rest or during simulated exercise. Therefore, the data obtained suggest that the reduction in exercise capacity following pF_RTN/C1_ inhibition is due to an inability to appropriately match minute ventilation to metabolic demands due to a loss of feedback control of tidal volume.

As arterial *P*O_2_, *P*CO_2_ and pH usually remain relatively constant^31^, the importance of the RTN in controlling ventilation during exercise may be due to its modulation of chemoreflex gain^51–53^. However, inhibition of the pF_RTN/C1_ region was found to reduce the pressor response to CO_2_, whilst the cardiovascular responses to simulated exercise were not affected. Furthermore, unlike whole body exercise (i.e., running), our experimental paradigm recruited only a limited number of leg muscles, and, therefore, was unlikely to cause major alterations in blood gases or concentration of other circulating humoral factors. Consequently, the reduction in ventilation during exercise in conditions of pF_RTN/C1_inhibition is unlikely to be due to disruption of chemosensory pathways. This opens up the possibility that RTN neurons may integrate other forms of afferent feedback that influence respiratory activity during exercise. For example, muscle metabotropic reflexes carried through somatic afferents may contribute to exercise-related increases in ventilation by increasing the chemoreflex gain of RTN neurons^54^.

In conclusion, the data obtained in this study suggest that whilst C1 neurons do not participate in orchestrating the cardiorespiratory response to exercise, the activity of RTN neurons is critically important. The RTN does not appear to be involved in triggering the initial increases in ventilation that pre-empt heightened metabolic demand at the onset of exercise. Instead, the activity of RTN neurons plays a critical role in maintaining increased respiratory effort during sustained exercise and, therefore, determines exercise capacity.

## Methods

Experiments were performed in 38 male Sprague-Dawley rats in accordance with the European Commission Directive 2010/63/EU (European Convention for the Protection of Vertebrate Animals used for Experimental and Other Scientific Purposes) and the United Kingdom Home Office (Scientific Procedures) Act (1986) with project approval from the Institutional Animal Care and Use Committee of the University College London.

### Surgery and microinjections of viral vectors

Young male Sprague-Dawley rats (50–100 g) were anesthetized with ketamine (60 mg·kg^−1^; i.m.) and medetomidine (250 μg·kg^−1^; i.m.) and supplemented with isofluorane as required. Animals were placed prone in a stereotaxic apparatus, with the nose bar 18 mm below the interaural line. Body temperature was maintained at 37 ± 0.5 °C. Bilateral microinjections of viral vectors (volume 250-350 nL per side) were placed stereotaxically into the pF_RTN/C1_: either 1 mm rostral, 1.7 mm lateral, and 3.6 mm ventral from the *calamus scriptorius* with the pipette holder arm set at 20°, or via two injections 1.7 mm lateral, and 3.6 mm ventral from the *calamus scriptorius*, with the pipette holder arm set at 16° and 20°. The wound was sutured and anaesthesia was reversed with atipamezole (1 mg·kg^−1^; i.m.). Postoperatively, the animals were treated with buprenorphine (0.1 mg·kg^−1^; i.m.) and carprofen (2 mg·kg^−1^) and were allowed to recover for 2–6 weeks.

### Viral vectors

Two AAV vectors were used in this study: AAV-2/2 hSyn-HA-hM_4_D(Gi)-IRES-mCitrine (HM_4_DR; 5.6 × 10^12^ vp·ml^−1^; UNC Vector Core), and AAV-2/2 hSyn-eGFP (eGFP; 4 × 10^12^ vp·ml^−1^; UNC Vector Core). Both of the viral vectors used the human synapsin promoter, and therefore predominantly transduced neurons showing higher tropism for the AAV 2/2 subtype, but not non-neuronal cells or neurons that do not show tropism for AAV 2/2 within the injection site, e.g., facial motoneurons.

### Exercise model

Exercise capacity of experimental rats was determined using a single lane rodent treadmill (Harvard Apparatus) as described in detail previously^55^. Experiments were conducted by an investigator blinded to the nature of the experimental groups. The distance covered by the animal was recorded and exercise capacity was expressed as work done in Joules (kg·m^−2^·s^−2^).

### Model of simulated exercise under anaesthesia

Rats were anaesthetized with urethane (1.2–1.7 g^−1^·kg^−1^; i.v.; following induction with 4% isoflurane). The brachial artery was catheterised to record arterial blood pressure. The trachea was cannulated to record respiratory flow using a pressure transducer. Expired O_2_ and CO_2_ were monitored online using a gas analyser. Body temperature was maintained at 37 ± 0.5 °C. Two protocols of simulated exercise were used: (1) rats were placed supine and the femoral nerves were stimulated bilaterally using bipolar silver electrodes (Fig. 1C); (2) rats were placed prone and the sciatic nerves stimulated bilaterally (Fig. 1C). Exposed nerves were submerged in mineral oil. Bladders were expressed before and during the experiment to minimize potential autonomic dysreflexia from bladder distension. Rats were given saline infusions when their bladder was expressed. Following preparative surgery, the animals were left to stabilize for 30 mins breathing room air.

Protocol 1: Femoral nerves were electrically stimulated (Pulses −100 ms; 6.5 V; 1 Hz; 10 min), with an alternating left-right pattern so that each leg stimulation was delayed 0.5 s in respect to the stimulations placed on the opposite side (Fig. 1C). Rats were allowed 40 mins to recover before they underwent femoral nerve stimulation for the second time. Following a 40 min recovery period, all animals were given clozapine-N-oxide (CNO, 2 mg·kg^−1^; I.P.), and the simulated exercise protocol was repeated 1 h after CNO administration.

Protocol 2: Sciatic nerves were stimulated in a similar manner to femoral nerves (Fig. 1C). The animals were allowed to recover for 40 min, after which they underwent sciatic nerve stimulation for the second time. Following another 40 min of recovery, a hypercapnic challenge was applied (9-10% CO_2_ in inspired air for 5 min). CNO (2 mg·kg^−1^; I.P.) was given 30 min after the hypercapnic challenge. The simulated exercise and hypercapnia protocols were repeated 1 h after CNO administration.

### Assessment of transgene expression in the brainstem

Rats were humanely killed by urethane overdose (>2 g·kg^−1^) and transcardially perfused with ice-cold (4 °C) paraformaldehyde solution (4% PFA). The brain was removed, postfixed overnight in PFA (4 °C), and cryoprotected in 30% sucrose. Brainstems were serially sectioned at 40 μm. Free-floating sections were incubated overnight in phosphate buffered saline (PBS) containing 0.1% Triton X-100 (PBT) and primary antibodies: chicken anti-green fluorescent protein (GFP; 1:500) and either goat anti-cholineacetyl transferase (ChAT; 1:100), or rabbit anti-tyrosine hydroxylase (TH; 1:250) antibody. Slices were washed in PBS (6 × 5 mins) and then incubated for 2–4 hrs in PBT containing secondary antibodies: either donkey anti-rabbit Alexa Fluor 568 (1:250), or donkey anti-Goat Alexa Fluor 568 (1:250) antibody. Slices were then washed in PBS (6 × 5 min) and incubated for 2–4 hours in a PBT containing goat anti-chicken Alexa Fluor 488 antibody (1:250). Slices were mounted, coverslipped and examined using a Leica fluorescent microscope with Q Capture acquisition software. To determine the efficacy of viral transfection and expression of HM_4_DR, cell counts of transduced neurons were performed in representative 40 µm sections throughout transduced brainstems. As the brains were not marked for left-right orientation and the injections were bilateral, the total number of neurons counted in each brain was divided by 2 to give the total number of neurons expressing the transgene per side. The numbers of neurons per side for each animal was averaged and provided in the text as means ± SEM.

### Data analysis

Power calculations were performed using Gpower 3 (www.ats.ucla.edu/stat/gpower/). Data were only included from animals where the pF_RTN/C1_ region was successfully transduced bilaterally, and no data were excluded from the analysis.

Exercise capacity was determined as work done (body weight x distance covered), expressed in kJ by multiplying by a conversion factor of 0.0098: exercise capacity (kJ) = body weight (kg) x distance covered (m) × 0.0098. The data obtained were found to be normally distributed (Shapiro–Wilk test; controls: p = 0.3; CNO: p = 0.2). The values of exercise capacity following saline or CNO injections in the same rats were compared using Student’s paired *t*-test. The baseline exercise capacity values in rats transduced to express HM_4_DR or eGFP were compared using Student’s unpaired *t*-test.

In anaesthetised rats, airflow pressure signals were amplified using the NeuroLog system connected to a 1401 interface and acquired on a computer using *Spike2* software (Cambridge Electronic Design). Airflow measurements were used to calculate: tidal volume (V_T_: signal trough at the end of expiration subtracted from the peak signal during inspiration, converted to mL following calibration), respiratory frequency (*f*: breaths per minute), and sigh rate (sighs per minute). Minute ventilation (V_e_) was calculated as V_T_ × *f*. As sighing leads to transient changes in both *f* and V_T_, which may skew the data in an unrepresentative manner, analysis was only performed on sigh-free periods of the recordings. Arterial blood pressure recordings were used to derive heart rate (HR: beats per minute) and to calculate mean arterial blood pressure (MAP: Diastolic pressure +1/3*[Systolic Pressure − Diastolic pressure]).

In the experiments involving simulated exercise, no differences in cardiorespiratory responses were found during stimulation of the femoral or sciatic nerves, thus the data from these groups were combined. Measurements (10 s) were taken at 4 time points: (i) at rest (baseline); (ii) during the initial response to nerve stimulation (Phase 1); (iii) near the end of the stimulation period (Phase 3); and iv) after the stimulation just before the re-appearance of inspiratory sighs. In the experiments involving CO_2_ challenges, cardiorespiratory measurements (10 s) were taken at 2 time points: (i) at rest; (ii) at the peak of the CO_2_ -induced respiratory response. The percentage change was calculated from these values (100*[value at the peak/value at rest]).

Statistical analysis was performed using OriginPro software. The data were tested for normality using Shapiro–Wilk test. All respiratory data from simulated exercise experiments were non-Gaussian (HM_4_DR transduced rats, V_T_, V_e_, sigh rate  – p < 0.001, and f – p = 0.002; eGFP transduced rats, V_T_, V_e_, sigh rate – p < 0.001, and f – p = 0.003), and were analyzed non-parametrically. Data were rank transformed and tested for significance using a 2-way repeated measures ANOVA with a Dunn-Sidak correction, and plotted as means ± standard error of the mean (SEM) of their ranks. All cardiovascular data obtained from simulated exercise paradigms were Gaussian (HM_4_DR transduced rats, HR – p = 0.2, and MAP – p = 0.5; eGFP transduced rats, HR – p = 0.2, and MAP – p = 0.3), and were tested parametrically. Data were tested using a 2-way repeated measures ANOVA with a Bonferroni correction, and are reported as means ± SEM.

All data obtained in the experiments involving CO_2_ challenges were normalized, treated as non-Gaussian and analyzed non-parametrically. Data were tested using 2-sided Wilcoxon signed-rank tests with a significance level of P < 0.05 and reported as median and IQR.

### Data Availability

The data that support the findings of this study are available from the corresponding authors upon reasonable request.
